# Implementation lessons learnt when trialling palliative care interventions in the intensive care unit: relationships between determinants, implementation strategies, and models of delivery—a systematic review protocol

**DOI:** 10.1186/s13643-022-02054-8

**Published:** 2022-09-02

**Authors:** S. A. Meddick-Dyson, J. W. Boland, M. Pearson, S. Greenley, R. Gambe, J. R. Budding, F. E. M. Murtagh

**Affiliations:** 1 Wolfson Palliative Care Research Centre, Hull York Medical School, University of Hull, Hull, HU6 7RX UK; 2 Cancer Awareness, Screening and Diagnostic Pathways Research Group (CASP), Hull York Medical School, University of Hull, Hull, UK; 3 Hull University Teaching Hospitals NHS Trust, Hull Royal Infirmary, Hull, UK

**Keywords:** Palliative care, Palliative medicine, Intensive care, Intensive care unit, Implementation science

## Abstract

**Background:**

Heterogeneity amongst palliative care interventions in the intensive care unit (ICU) and their outcomes has meant that, even when found to be effective, translation of evidence into practice is hindered. Previous evidence reviews have suggested that the field of ICU-based palliative care would benefit from well-designed, targeted interventions, with explicit knowledge translation research demonstrating valid implementation strategies. Reviewing effectiveness studies alongside process evaluations for these interventions will give insight into the implementation barriers or constraints identified, and the implementation strategies adopted.

**Methods:**

A systematic review to identify and synthesise knowledge on how models of integrating palliative care into the ICU have been implemented and provide critical recommendations for successful future development and implementation of complex interventions in the field. The search will be carried out using MEDLINE, Embase, Cochrane, CINAHL, and PsycINFO. The search strategy will combine terms related to palliative care, intensive care, and implementation. Only full-text articles will be considered and conference abstracts excluded. There will be no date or language restrictions. The Implementation Research Logic Model will be used as a framework for synthesis. Findings will be reported following the Preferred Reporting Items of Systematic Reviews and Meta-Analyses (PRISMA) guidelines.

**Discussion:**

This review will provide understanding of implementation facilitators, barriers, and strategies, when employing palliative care interventions within the ICU. This will provide valuable recommendations for successful future development of complex interventions using implementation frameworks or theories. This can increase the potential for sustained change in practice, reduce heterogeneity in interventions, and therefore help produce measurable and comparable outcomes.

**Systematic review registration:**

International Prospective Register of Systematic reviews PROSPERO (CRD42022311052)

**Supplementary Information:**

The online version contains supplementary material available at 10.1186/s13643-022-02054-8.

## Background

Implementation research is defined as “the scientific study of methods to promote the systematic uptake of research findings and other evidence-based practices into routine practice, and, hence, to improve the quality and effectiveness of health services” [1, 2]. Evidence-based interventions are often found to be effective at improving health outcomes, behaviours, and/or health related environments [3, 4], but there is a historic gap between this evidence base and getting evidence into practice. The focus has frequently been on conducting intervention studies, rather than researching whether and how the findings translate into health impact [2].

Intensive care units (ICUs) deliver specialised care to critically ill patients with life-threatening conditions with the primary goal to prevent further physiological deterioration while the underlying dysfunction is treated [5, 6]. For patients with life-threatening illness, palliative care encompasses complex symptom control, communication surrounding care and treatments, addressing patient values, transitional planning, and support for those around them [7]. It achieves these goals through early recognition, detailed assessment, and treatment of physical, psychosocial, and/or spiritual needs [7]. Although survival in ICUs has improved with clinical and technological advances over time, 15–20% of intensive care patients will die during their hospital admission [8]. Time in the ICU can be fraught with burdensome symptoms and difficult discussions and decisions for patients and their families [9]. Hence, it is important to have high quality and effective palliative and end-of-life care within ICUs and the Faculty of Intensive Care Medicine, amongst other professional bodies, recommends this [10–12]. The faculty states that end-of-life care remains a necessary core skill set for critical care teams: strengthening inter-personal relationships between patients and those close to them by providing a sense of control, minimising distress, alleviating both psychological and physical burdens, meeting spiritual needs, and understanding legal and ethical principles, amongst other benefits [10]. An international consensus conference held in Belgium identified a number of concerns with end-of-life care in the ICU, including terminology used, variability, communication issues, and determining preferences [12]. The jury strongly recommended that research be conducted to improve end-of-life care [13]. In 2001, an expert group convenes to develop a research agenda for end-of-life care in the ICU, and amongst their priorities were as follows: addressing the cultural chasm between clinicians in the ICU, educating the public and providers, developing innovative strategies to improve quality of care, and structural and organisational changes [14]. These mirror the aims of implementation science research.

Two models are commonly used to exemplify how palliative care can be integrated into the ICU [15]. The “consultative model” promotes involvement of and consultation with specialist palliative care teams, especially for patients at high risk of a poor outcome, while the “integrative model” aims to support intensive care teams to incorporate palliative care into their daily practice [15]. These two models denote each end of a spectrum rather than being mutually exclusive, and practice may see interventions favouring one approach over the other, or a hybrid of the two.

A recent systematic review of randomised clinical trials and observational studies reported palliative care interventions within the ICU setting, assessed their potential impact on ICU practice, and evaluated differences in palliative care approaches between different countries [16]. Implementation of these interventions was not assessed. This review concluded that the field of ICU-based palliative care would benefit from well-designed, targeted interventions [16]. Implementation research can help achieve this. An earlier systematic review synthesised studies of the experiences and perceptions of health care professionals in adopting palliative care interventions in ICUs [17]. However, it did not include effectiveness studies and so did not establish barriers or facilitators to implementation or report any implementation strategies used by researchers. It concluded that we need explicit knowledge translation research demonstrating valid implementation strategies [17]. Reviewing effectiveness studies alongside process evaluations for palliative care interventions within intensive care will give valuable insights into implementation barriers or constraints identified and demonstrate the implementation strategies that have been found to complement or overcome them. Moreover, it will help gain insight into implementation strategies that have been tried and found to be ineffective.

## Aim

Using a logic model as a framework for synthesis, this review will aim to identify and synthesise knowledge on how models of integrating palliative care into the ICU have been implemented and provide critical recommendations for successful future development and implementation of complex interventions in the field. The resulting high-quality interventions to improve palliative care on the ICU will benefit patients, their families and carers, and health care professionals.

## Objectives

- To identify and describe evidence on facilitators of, or constraints on, implementation of palliative care interventions within the ICU.
- To identify and describe any specific implementation strategies reported, that have been used to address facilitators or constraints, when employing palliative care interventions within the ICU.
- To explore the effect of these strategies on implementation and outcomes.
- To identify and describe differences in implementation when looking at palliative care interventions that are characterised as integrative or consultative.

## Methods

### Data sharing

This systematic review protocol was registered with the PROSPERO International Prospective Register of Systematic Reviews (CRD42022311052) before searches were carried out and will be reported using Preferred Reporting Items of Systematic Reviews and Meta-Analyses (PRISMA) [18].

### Eligibility criteria

The eligibility criteria is shown in Table 1.

**Table 1:** Inclusion and exclusion criteria

Inclusion	Exclusion
*Population:* Adult patients admitted to the ICU or HDU and/or their families AND/OR palliative care professionals or teams	Studies solely considering children and adolescents (defined as those aged ≤ 18)
*Intervention:* Palliative care intervention occurring in/in relation to the ICU	
*Comparator:* No palliative care intervention or, to include studies that compare separate palliative care interventions; alternative palliative care intervention(s)	
*Outcomes:* Any of the following implementation outcomes; acceptability, adoption, appropriateness, costs, feasibility, fidelity, penetration, sustainability AND/OR any system/content/clinical/patient and family related palliative care outcomes	Studies with no reported outcomes Studies with no information of implementation
*Study design:* Controlled trials (randomised and non-randomised including observational studies). Process evaluations (quantitative, qualitative or mixed-methods), conducted either alongside a comparative study or stand-alone	Case reports or series Editorials/commentaries Opinion papers Publications only as abstracts Review papers
*Dates:* All dates	
*Language:* Not limited to language or location	

### Clarification of terms

Metaxa et al. defined a palliative care intervention as one that was aimed at improving the quality of life of at-risk-of-dying patients and/or their families [16]. This review will utilise the same definition. As previously mentioned, the integrative and consultative models of integrating palliative care into the ICU are not mutually exclusive, but a dichotomy is useful for comparative research and so will be used for the purpose of this review. For consistency, the dichotomy used by Metaxa et al. will be used, and mixed interventions or those involving consultations with palliative care-trained specialists or ethicists, will be classified as consultative [16].

For the population stated in Table 1, families are defined as individuals who provide support and with whom the patient has a significant relationship according to the definition developed by a guideline writing committee for family-centred care in the ICU [19]. They may be related or unrelated to the patient [19]. This mirrors the systematic review by Metaxa et al. [16].

Implementation strategies refer to “methods or techniques used to enhance the adoption, implementation, and sustainability” of an evidence-based intervention [4, 20]. Determinants are the modifiable factors that the implementation strategy aims to change to influence implementation of evidence-based interventions [4, 21], in other words factors that facilitate or constrain implementation.

### Information sources and search strategy

This review aims to include the comparative studies identified in Metaxa et al.’s paper that give information regarding implementation, as well as additional studies looking at palliative care interventions relating to the ICU or comparing multiple palliative care interventions. It will also include process evaluations conducted either alongside a comparative study or stand-alone to capture information on implementation.

The search strategy, developed with an information specialist (SG), draws on relevant primary evidence and reviews to refine search terms.

- ◦ MEDLINE, EMBASE and PsycINFO via OVID, CINAHL via EbscoHost, CENTRAL and Cochrane Database of Systematic Reviews via The Cochrane Library, databases will be used.
- ◦ No date restrictions on year of publication to maximise yield.
- ◦ Not limited to language or location to maximise generalisability.
- ◦ Reference lists of all included papers and any relevant reviews will be hand searched.
- ◦ Searches will use database appropriate Subject Headings terms (e.g. MeSH) and free text terms, combining adaptations of the searches from Metaxa et al. [16] and novel strategies (see Appendix 1 for example search strategy for MEDLINE).
- ◦ The results from combining the following three concepts (line 81 of MEDLINE search):
- Terms related to palliative and end of life services (adapted existing)
- *AND*
- Terms related to intensive and critical care (adapted existing)
- *AND*
- Terms related to implementation science (developed for this review)
- ◦ Will be pooled using the ‘OR’ syntax with the following three concepts (line 82 of MEDLINE search):
- Terms related to palliative and end of life services
- *AND*
- Terms related to intensive and critical care
- *AND*
- Terms to identify controlled studies (developed for this review)

Following guidance from the Cochrane Handbook for Systematic Reviews of Interventions, process evaluations will be identified using two methods:

- Search filters related to process evaluations will be added to the implementation science search concept (shown in Appendix 1).
- Forwards and backwards citation searching using Citation Indexes will be conducted on included papers describing controlled trials to identify linked reports. The citations will be screened at the beginning of data extraction by the same reviewer. Any identified process evaluations will be included in the data extraction process for the paired study [22].
- Searches will be re-run December 2022 to ensure they are updated.

### Study selection

- ◦ Identified studies will be managed in EndNote and duplicate references will be removed before screening.
- ◦ Google Translate will be used to aid in screening for any papers not written in English. Manual translation will be used to enable accurate extraction if the papers are selected.
- ◦ Title and abstracts will be screened to identify primary studies or process evaluations that meet the inclusion and exclusion criteria (see Table 1). Two reviewers (SMD and RG) will consider the application of the criteria independently. All studies will be dual screened. Covidence software will be used. Conflicts will be resolved via discussion between the reviewing authors. A third author (FM or JWB) will be consulted if no consensus is reached.
- ◦ The full-texts of potentially eligible papers will be further screened in the same way.
- ◦ If an informed inclusion/exclusion decision cannot be made from the title and abstract alone then the study will be taken to full text screening.
- ◦ If full text is not available after contacting the author, then the study will not be included in the review.

### Data extraction

- ◦ A predefined and piloted data extraction form (see Additional file 1) designed for this review using the Implementation Research Logic Model as a framework will be used.
- ◦ Study data will be extracted on Covidence by JRB and implementation data extracted by SMD. All data extracted will be checked by the second reviewer to ensure consensus. Conflicts will be resolved via discussion between the reviewing authors. A third reviewer will be consulted if no consensus is reached (FM).
- ◦ Variables to be extracted will include:
  1. Study data: Reference details (citation to include year of publication, first author name, journal), study design, data type.
  2. Participant data: population, setting, country, group information.
  3. Comparator(s).
  4. Palliative care intervention data: Headings as per the Template for Intervention Description and Replication (TIDieR) [23]. Classification domain according to the Metaxa et al. system [16]. Whether integrative or consultative model used.
  5. Determinants of interventions using Consolidated Framework for Integration Research headings [24].
  6. Implementation strategies resulting from the Expert Recommendations for Implementing Change study [20].
  7. Implementation and/or palliative care outcome data; outcome definition, category, and measure. Effectiveness of interventions on palliative care outcomes is outside of the scope of this study, and so this data will not be collected.
- ◦ Reviewers will identify and comment on factors enabling and constraining implementation and implementation strategies that have been formally reported or informally reflected on within the paper.
- ◦ Where the study data is missing, we will attempt to contact the corresponding author.

### Data synthesis

**Fig. 1:**
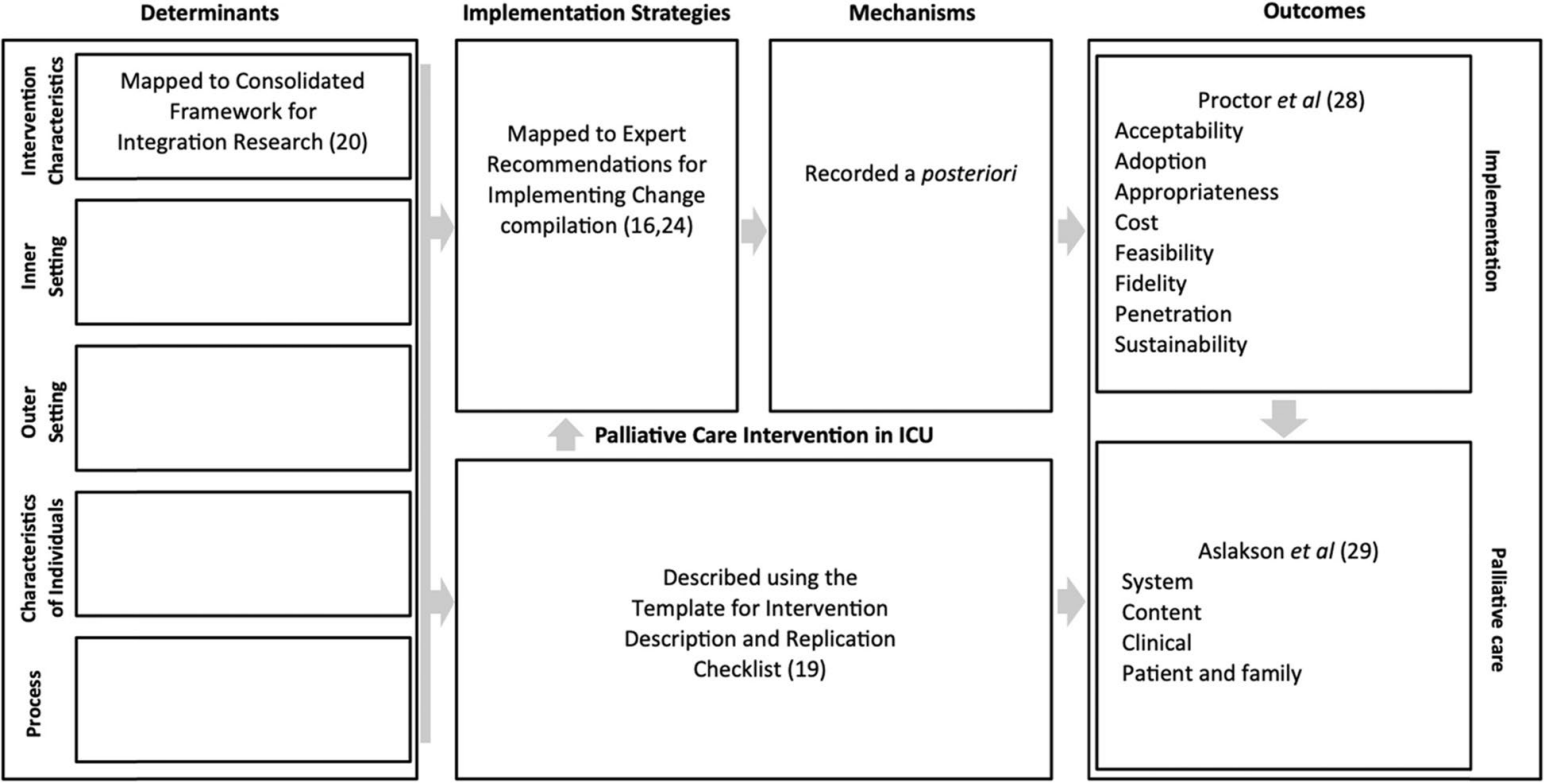
Implementation Research Logic Model schematic (adapted from Smith et al.)

Study characteristics will be described using an initial descriptive synthesis. The Implementation Research Logic Model will be used to index predefined concepts and interpret relationships between them. The Implementation Research Logic Model is a process-orientated logic model described in implementation science literature [25]. Logic models can provide scaffolding to integrate the findings of varying evidence [22, 26]. A priori logic models are being increasingly used in systematic reviews [27]. They can be deconstructed for data extraction and then reconstructed to show relationships between components [26]. The Implementation Research Logic Model will be used as it gives a clear format to present and examine relationships between the components that this review is aiming to explore implementation determinants, implementation strategies, mechanisms, palliative care interventions, and implementation and clinical outcomes (see Fig. 1) [25].

- ◦ Determinants: The model draws the domain names for determinants from the Consolidated Framework for Implementation Research: process, characteristics of individuals, outer setting, inner setting, and intervention characteristics [24].
- ◦ Implementation strategies: The Expert Recommendations for Implementing Change study involved self-identified implementation experts selecting implementation strategies most likely to address each Consolidated Framework for Integration Research barrier [20]. Seventy-three Expert Recommendations for Implementing Change strategies were identified and further characterised into 9 subheadings: engage consumers, use evaluative and iterative strategies, change infrastructure, adapt and tailor to the context, develop stakeholder interrelationships, utilise financial strategies, support clinicians, provide interactive assistance, and train and educate stakeholders [20, 28]. The supplementary material to aid use of the Implementation Research Logic Model suggests use of this taxonomy for implementation strategies [25].
- ◦ Mechanisms: Mechanisms are described as the processes or events through which implementation outcomes are effected by implementation strategies [29]. Current literature describes a lack of conceptual, theoretical, and empirical work articulating mechanisms for the Expert Recommendations for Implementing Change strategies and identifying measures of such [30]. No predefined list of mechanisms exists for use, therefore any mechanisms identified will be recorded a posteriori.
- ◦ Palliative care interventions: Interventions will be described using the Template for Intervention Description and Replication (TIDieR) [23]. It is a comprehensive framework developed to improve the completeness of reporting of interventions and has been recommended for use in systematic reviews [23, 31]. Interventions will be classified using a new system recommended by Metaxa et al. where palliative care interventions on ICU are split into five categories: communication interventions, ethics consultations, educational interventions, palliative care team involvement, and advance care planning [16].
- ◦ Outcomes:
  - ◦ Implementation: The format of the implementation outcomes within the Implementation Research Logic Model is taken from a predefined list of outcomes: acceptability, adoption, appropriateness, costs, feasibility, fidelity, penetration, and sustainability [32].
  - ◦ Palliative care: A review aiming to provide a conceptual framework for palliative care outcomes in ICU reflected on work looking at palliative care outcomes as well as published interventions focused on palliative care in the ICU and the outcomes utilised when reporting these [33]. They established that the outcomes could be conceptualised into four groups: system/content/clinical/patient and family related [33]. These groups were also referenced Metaxa *et al*. and will be used in this review [16].

Evidence not captured by the framework will be analysed using the principles of thematic analysis to generate themes. To include quantitative data, findings will be transformed and integrated following JBI guidance of the convergent integrated approach [34].

### Assessment of the quality of included studies

- ◦ The methodological quality of included studies and process evaluations will be assessed using the Mixed Methods Appraisal Tool [35]. The Mixed Methods Appraisal Tool has been assessed for reliability and efficiency and offers a comprehensive way to assess studies of numerous method types and produces an overall score as a percentage [36]. This allows the same tool to be used across the review (see Appendix 2).
- ◦ Papers will be included regardless of their quality, but this will be considered and commented on when interpreting findings.

### Confidence in cumulative evidence

The strength of the body of evidence will be assessed using the Grading of Recommendations Assessment, Development, and Evaluation - Confidence in the Evidence from Reviews of Qualitative research (GRADE-CERQual) [37]. Any amendments to the protocol will be clearly stated within the final manuscript methods section.

## Discussion

High-levels of diversity has been documented amongst interventions to integrate and improve palliative care within the ICU [16]. When reviewed by Metaxa et al., interventions could be grouped into (i) communication interventions, (ii) ethics consultations, (iii) educational interventions, (iv) palliative care team involvement, or (v) advance care planning; however, similar interventions within these groups differed significantly in aspects such as delivery model or ‘dose’ [16]. Interventions produced heterogeneous outcome measures that have not allowed for quantitative meta-analysis [16]. Only a minority of studies trialled specific, well-defined interventions [16]. This variability across interventions, their outcomes, and the fact that effectiveness trials themselves do not tend to translate to the intervention being implemented or sustained in practice [2] means that there is a grave potential for research waste developing interventions in the future.

Understanding strategies to promote sustained uptake of effective interventions into practice via implementation science can address this. There is not currently a systematic review which synthesises evidence on what aids implementation or hinders it. This systematic review will synthesise current available evidence on how models of integrating palliative care into the ICU have been implemented. Using a logic model as a framework for this synthesis, it will identify any specific implementation strategies that have been used to address facilitators or constraints when employing palliative care interventions within the ICU. This will provide critical recommendations for successful future development of complex interventions using implementation frameworks or theories. This can increase the potential for sustained evidence-based change in practice, and therefore improve outcomes for patients and families in ICU.

### Supplementary Information

**Additional file 1.** Data Extraction Form.

## Data Availability

Not applicable.

## Appendix 1

### Example search strategy

#### Ovid MEDLINE(R) ALL <1946 to January 19, 2022>

1. “burn* unit*”.ti,ab.
2. “coronary care unit*”.ti,ab.
3. “respiratory care unit*”.ti,ab.
4. “intensive cardiac care”.ti,ab.
5. icu.ti,ab.
6. Intensive Care Units/
7. “intensive care unit*”.ti,ab.
8. “high dependency unit*”.ti,ab.
9. hdu.ti,ab.
10. Burn Units/
11. Coronary Care Units/
12. “continuous renal replacement therapy”.ti,ab.
13. Respiratory Care Units/
14. “critically ill”.ti,ab,kw. or critical illness/
15. or/1-14 [ICU terms]
16. Palliative Care/
17. Palliative Medicine/
18. (hospice and palliative care nursing).mp.
19. Terminal Care/
20. Hospice Care/
21. palliat*.ti,ab.
22. “eol care”.ti,ab.
23. EOLC.ti,ab.
24. (“ethic* consultat*” or “ethics intervention*”).mp.
25. “terminal care”.ti,ab.
26. (“end of life” or end-of-life).ti,ab,kf.
27. “terminal illness*”.ti,ab.
28. “terminal patient*”.ti,ab.
29. “terminally ill”.ti,ab.
30. “limited survival”.ti,ab.
31. “advance* care plan*”.mp.
32. “terminal phase”.ti,ab.
33. “terminal stage*”.ti,ab.
34. “life-limiting”.ti,ab.
35. “comfort care”.ti,ab.
36. “symptom management”.ti,ab.
37. “symptomatic treatment”.ti,ab.
38. “symptomatic therapy”.ti,ab.
39. “limited life”.ti,ab.
40. “supportive care”.ti,ab.
41. “supportive treatment”.ti,ab.
42. “supportive therapy”.ti,ab.
43. (“high risk of death” or “family support” or bereavement).mp.
44. or/16-43 [palliative terms]
45. Implementation Science/ or implementation.ti,ab,kw.
46. facilitat*.mp.
47. barrier*.mp.
48. determinant*.mp.
49. implement*.mp.
50. integrat*.mp.
51. disseminat*.mp.
52. knowledge translation.mp.
53. adhere*.mp.
54. adopt*.mp.
55. compliance.mp.
56. (process adj evaluation*).mp.
57. acceptability.mp.
58. Feasibility Studies/ or feasibility.mp.
59. sustainability.mp.
60. Process Assessment, Health Care/ or “Outcome and Process Assessment, Health Care”/
61. Program Evaluation/
62. (intervention or quality improvement* or stakeholder*).mp.
63. or/45-62 [implementation terms]
64. exp Case-Control Studies/
65. exp Cohort Studies/
66. Randomized Controlled Trials as Topic/
67. randomised controlled trial.mp.
68. exp controlled clinical trial/
69. cohort study.mp.
70. case-control study.mp.
71. observational study.mp. or Observational Study/ or Controlled Before-After Studies/ or Comparative Study/
72. 64 or 65 or 66 or 67 or 68 or 69 or 70 or 71 [Controlled studies]
73. Communication/ or communication.ti,kf.
74. cpr.mp. or Cardiopulmonary Resuscitation/
75. Decision Making/
76. decision making.mp.
77. Cardiopulmonary Resuscitation.mp.
78. 74 or 75 or 76 or 77
79. 73 and 78 [communication around decisions/CPR]
80. 44 or 79 [palliative or communication around decisions/CPR]
81. 15 and 80 and 63 [ICU and pall/communication and implementation]
82. 15 and 80 and 72 [ICU and pall/communication and controlled studies]
83. limit 82 to dt=20200801-20220224 [limit controlled studies to after Metaxa search date]
84. 81 or 83
85. Case Reports/
86. Editorial/
87. Comment/
88. (adolescent/ or exp Pediatrics/ or exp child/ or exp infant/) not exp Adult/
89. (animals not humans).sh.
90. or/85-89 [unwanted publications and participants]
91. 84 not 90 [Final]

## Abbreviations

GRADE-CERQual: Grading of Recommendations Assessment, Development, and Evaluation - Confidence in the Evidence from Reviews of Qualitative research
ICU: Intensive care unit
PRISMA: Preferred Reporting Items of Systematic Reviews and Meta-Analyses
TIDieR: Template for Intervention Description and Replication
