# Supplementary material for: Implementation lessons learnt when trialling palliative care interventions in the intensive care unit: relationships between determinants, implementation strategies, and models of delivery—a systematic review protocol
Source: Syst Rev. 2022 Sep 2;11:186. doi: 10.1186/s13643-022-02054-8 (PMC9438136; doi:10.1186/s13643-022-02054-8)
Supplement: Supplementary file 1 — Additional file 1. Data Extraction Form. [file 13643_2022_2054_MOESM1_ESM.docx]

**Additional file 1: Data Extraction Form**

**DATA EXTRACTION FORM**

| **Study ID *(surname of first author and year first full report of study was published e.g. Smith 2001)*** | | | | | | | | | |
| --- | --- | --- | --- | --- | --- | --- | --- | --- | --- |
|  | | | | | | | | | |
| **Date form completed** | | | | | | | | | |
|  | | | | | | | | | |
| **ID person extracting data** | | | | | | | | | |
|  | | | | | | | | | |
| 1. **Study data** | | | | | | | | | |
| 1.1 Reference details (citation to include year of publication, first author name, journal) | | | | | | | | | |
| Study reference | | | | | Process evaluation reference (if applicable) | | | | |
|  | | | | |  | | | | |
| 1.2 Study design | |  | | | | | | | |
| 1. **Participants** | | | | | | | | | |
| **P**ICOS**:** Adult patients admitted to the ICU or HDU and/or their families AND/OR palliative care professionals or teams | | | | | | | | | |
| 2.1 Clinical setting | |  | | | | | | | |
| 2.2 Country of study | |  | | | | | | | |
| 2.3 Population description | |  | | | | | | | |
| 2.4 Total no. participants and group numbers | | Intervention study | | | | | Implementation study | | |
|  |  |  | | | | |  | | |
| 1. **Comparator(s)** | | | | | | | | | |
| PI**C**OS**:** No palliative care intervention or alternative palliative care intervention(s) | | | | | | | | | |
| 3.1 Type of comparator, if any (e.g. usual care) | |  | | | | | | | |
| 1. **Intervention(s)** (Taken from TIDieR Checklist) [23] | | | | | | | | | |
| P**I**COS**:** Palliative care intervention occurring in/in relation to the ICU | | | | | | | | | |
| 4.1 Brief Name | | | | | | | | | |
|  | | | | | | | | | |
| 4.2 Why | | | | | | | | | |
|  | | | | | | | | | |
| 4.3 What (Materials and procedures) | | | | | | | | | |
|  | | | | | | | | | |
| 4.4 Who provided | | | | | | | | | |
|  | | | | | | | | | |
| 4.5 How (Mode of delivery) | | | | | | | | | |
|  | | | | | | | | | |
| 4.6 Where | | | | | | | | | |
|  | | | | | | | | | |
| 4.7 When and how much | | | | | | | | | |
|  | | | | | | | | | |
| 4.8 Tailoring | | | | | | | | | |
|  | | | | | | | | | |
| 4.9 Modifications | | | | | | | | | |
|  | | | | | | | | | |
| 4.10 How well | | | | | | | | | |
|  | | | | | | | | | |
| 4.11 Actual | | | | | | | | | |
|  | | | | | | | | | |
| 4.12 Domain |  | | | 4.13 Integrative/Consultative | | | | | |
| 1. **Determinants** (Consolidated Framework for Integration Research) [24] | | | | | | | | | |
| **5.1 Intervention Characteristics** | | | | | | | | | |
| 5.1.1 Intervention Source | |  | | | | | | | |
| 5.1.2 Evidence Strength & Quality | |  | | | | | | | |
| 5.1.3 Relative Advantage | |  | | | | | | | |
| 5.1.4 Adaptability | |  | | | | | | | |
| 5.1.5 Trialability | |  | | | | | | | |
| 5.1.6 Complexity | |  | | | | | | | |
| 5.1.7 Design Quality & Packaging | |  | | | | | | | |
| 5.1.8 Cost | |  | | | | | | | |
| **5.2 Outer setting** | | | | | | | | | |
| 5.2.1 Patient Needs & Resources | |  | | | | | | | |
| 5.2.2 Cosmopolitanism | |  | | | | | | | |
| 5.2.3 Peer Pressure | |  | | | | | | | |
| 5.2.4 External Policy & Incentives | |  | | | | | | | |
| **5.3 Inner setting** | | | | | | | | | |
| 5.3.1 Structural Characteristics | |  | | | | | | | |
| 5.3.2 Networks & Communications | |  | | | | | | | |
| 5.3.3 Culture | |  | | | | | | | |
| 5.3.4 Implementation Climate | |  | | | | | | | |
| 5.3.5 Tension for Change | |  | | | | | | | |
| 5.3.6 Compatibility | |  | | | | | | | |
| 5.3.7 Relative Priority | |  | | | | | | | |
| 5.3.8 Organizational Incentives & Rewards | |  | | | | | | | |
| 5.3.9 Goals and Feedback | |  | | | | | | | |
| 5.3.10 Learning Climate | |  | | | | | | | |
| 5.3.11 Readiness for Implementation | |  | | | | | | | |
| 5.3.12 Leadership Engagement | |  | | | | | | | |
| 5.3.13 Available Resources | |  | | | | | | | |
| 5.3.14 Access to Knowledge & Information | |  | | | | | | | |
| 5.3.15 Structural Characteristics | |  | | | | | | | |
| **5.4 Characteristics of individuals** | | | | | | | | | |
| 5.4.1 Knowledge & Beliefs about the Intervention | |  | | | | | | | |
| 5.4.2 Self-efficacy | |  | | | | | | | |
| 5.4.3 Individual Stage of Change | |  | | | | | | | |
| 5.4.4. Individual Identification with Organization | |  | | | | | | | |
| 5.4.5 Other Personal Attributes | |  | | | | | | | |
| **5.5 Process** | | | | | | | | | |
| 5.5.1 Planning | |  | | | | | | | |
| 5.5.2 Engaging | |  | | | | | | | |
| 5.5.3 Opinion Leaders | |  | | | | | | | |
| 5.5.4 Formally Appointed Internal Implementation Leaders | |  | | | | | | | |
| 5.5.5 Champions | |  | | | | | | | |
| 5.5.6 External Change Agents | |  | | | | | | | |
| 5.5.7 Executing | |  | | | | | | | |
| 5.5.8 Reflecting & Evaluating | |  | | | | | | | |
| 1. **Implementation strategies** (Expert Recommendations for Implementing Change) [20, 28] | | | | | | | | | |
| **6.1 Engage consumers** | | | | | | | | | |
| 6.1.1 Involve patients/consumers and family members | | | | |  | | | | |
| 6.1.2 Intervene with patients/consumers to enhance uptake and adherence | | | | |  | | | | |
| 6.1.3 Prepare patients/consumers to be active participants | | | | |  | | | | |
| 6.1.4 Increase demand | | | | |  | | | | |
| 6.1.5 Use mass media | | | | |  | | | | |
| **6.2 Use evaluative and iterative strategies** | | | | | | | | | |
| 6.2.1 Assess for readiness and identify barriers and facilitators | | | | |  | | | | |
| 6.2.2 Audit and provide feedback | | | | |  | | | | |
| 6.2.3 Purposefully reexamine the implementation | | | | |  | | | | |
| 6.2.4 Develop and implement tools for quality monitoring | | | | |  | | | | |
| 6.2.5 Develop and organize quality monitoring systems | | | | |  | | | | |
| 6.2.6 Develop a formal implementation blueprint | | | | |  | | | | |
| 6.2.7 Conduct local need assessment | | | | |  | | | | |
| 6.2.8 Stage implementation scale up | | | | |  | | | | |
| 6.2.9 Obtain and use patients/consumers and family feedback | | | | |  | | | | |
| 6.2.10 Conduct cyclical small tests of change | | | | |  | | | | |
| **6.3 Change infrastructure** | | | | | | | | | |
| 6.3.1 Mandate change | | | | |  | | | | |
| 6.3.2 Change record systems | | | | |  | | | | |
| 6.3.3 Change physical structure and equipment | | | | |  | | | | |
| 6.3.4 Create or change credentialing and/or licensure standards | | | | |  | | | | |
| 6.3.5 Change service sites | | | | |  | | | | |
| 6.3.6 Change accreditation or membership requirements | | | | |  | | | | |
| 6.3.7 Start a dissemination organization | | | | |  | | | | |
| 6.3.8 Change liability laws | | | | |  | | | | |
| **6.4 Adapt and tailor to the context** | | | | | | | | | |
| 6.4.1 Tailor strategies | | | | |  | | | | |
| 6.4.2 Promote adaptability | | | | |  | | | | |
| 6.4.3 Use data experts | | | | |  | | | | |
| 6.4.4 Use data warehousing techniques | | | | |  | | | | |
| **6.5 Develop stakeholder interrelationships** | | | | | | | | | |
| 6.5.1 Identify and prepare champions | | | | |  | | | | |
| 6.5.2 Organize clinician implementation team meetings | | | | |  | | | | |
| 6.5.3 Recruit, designate, and train for leadership | | | | |  | | | | |
| 6.5.4 Inform local opinion leaders | | | | |  | | | | |
| 6.5.5 Build a coalition | | | | |  | | | | |
| 6.5.6 Obtain formal commitments | | | | |  | | | | |
| 6.5.7 Identify early adopters | | | | |  | | | | |
| 6.5.8 Conduct local consensus discussions | | | | |  | | | | |
| 6.5.9 Capture and share local knowledge | | | | |  | | | | |
| 6.5.10 Use advisory boards and workgroups | | | | |  | | | | |
| 6.5.11 Use an implementation advisor | | | | |  | | | | |
| 6.5.12 Model and simulate change | | | | |  | | | | |
| 6.5.13 Visit other sites | | | | |  | | | | |
| 6.5.14 Involve executive boards | | | | |  | | | | |
| 6.5.15 Develop an implementation glossary | | | | |  | | | | |
| 6.5.16 Develop academic partnerships | | | | |  | | | | |
| 6.5.17 Promote network weaving | | | | |  | | | | |
| **6.6 Utilize financial strategies** | | | | | | | | | |
| 6.6.1 Fund and contract for the clinical innovation | | | | |  | | | | |
| 6.6.2 Access new funding | | | | |  | | | | |
| 6.6.3 Place innovation on fee for service lists/formularies | | | | |  | | | | |
| 6.6.4 Alter incentive/allowance structures | | | | |  | | | | |
| 6.6.5 Make billing easier | | | | |  | | | | |
| 6.6.6 Alter patient/consumer fees | | | | |  | | | | |
| 6.6.7 Use other payment schemes | | | | |  | | | | |
| 6.6.8 Develop disincentives | | | | |  | | | | |
| 6.6.9 Use capitated payments | | | | |  | | | | |
| **6.7 Support clinicians** | | | | | | | | | |
| 6.7.1 Facilitate relay of clinical data to providers | | | | |  | | | | |
| 6.7.2 Remind clinicians | | | | |  | | | | |
| 6.7.3 Develop resource sharing agreements | | | | |  | | | | |
| 6.7.4 Revise professional roles | | | | |  | | | | |
| 6.7.5 Create new clinical teams | | | | |  | | | | |
| **6.8 Provide interactive assistance** | | | | | | | | | |
| 6.8.1 Facilitation | | | | |  | | | | |
| 6.8.2 Provide local technical assistance | | | | |  | | | | |
| 6.8.3 Provide clinical supervision | | | | |  | | | | |
| 6.8.4 Centralize technical assistance | | | | |  | | | | |
| **6.9 Train and educate stakeholders** | | | | | | | | | |
| 6.9.1 Conduct ongoing training | | | | |  | | | | |
| 6.9.2 Provide ongoing consultation | | | | |  | | | | |
| 6.9.3 Develop educational materials | | | | |  | | | | |
| 6.9.4 Make training dynamic | | | | |  | | | | |
| 6.9.5 Distribute educational materials | | | | |  | | | | |
| 6.9.6 Use train-the-trainer strategies | | | | |  | | | | |
| 6.9.7 Conduct educational meetings | | | | |  | | | | |
| 6.9.8 Conduct educational outreach visits | | | | |  | | | | |
| 6.9.10 Create a learning collaborative | | | | |  | | | | |
| 6.9.11 Shadow other experts | | | | |  | | | | |
| 6.9.12 Work with educational institutions | | | | |  | | | | |
| 1. **Outcome(s)/Finding(s)** | | | | | | | | | |
|  | | 7.1 Palliative care (PC) outcome | PC outcome(s) category (patient/family, clinician, system, content related) | | | 7.2 Implementation outcome(s) | | | Implementation outcome category (acceptability, adoption, appropriateness, costs, feasibility, fidelity, penetration, sustainability) |
| Primary outcome(s) + unit of measurement | |  |  | | |  | | |  |
| Secondary outcome(s) + unit of measurement | |  |  | | |  | | |  |
| 1. **Key themes identified outside of framework** | | Theme or subtheme | | | | | | Illustration from publication | |
|  |  |  | | | | | |  | |
| Author’s conclusion | |  | | | | | | | |
| Reviewer’s comments | |  | | | | | | | |
| Other information | |  | | | | | | | |
